# Supplementary material for: The causal relationship between the human gut microbiota and pyogenic arthritis: a Mendelian randomization study
Source: Front Cell Infect Microbiol. 2024 Nov 26;14:1452480. doi: 10.3389/fcimb.2024.1452480 (PMC11629706; doi:10.3389/fcimb.2024.1452480)
Supplement: Supplementary file 1 [file DataSheet1.zip › mendelian test/ebi-a-GCST90027579.csv_leave_one_out.pdf]

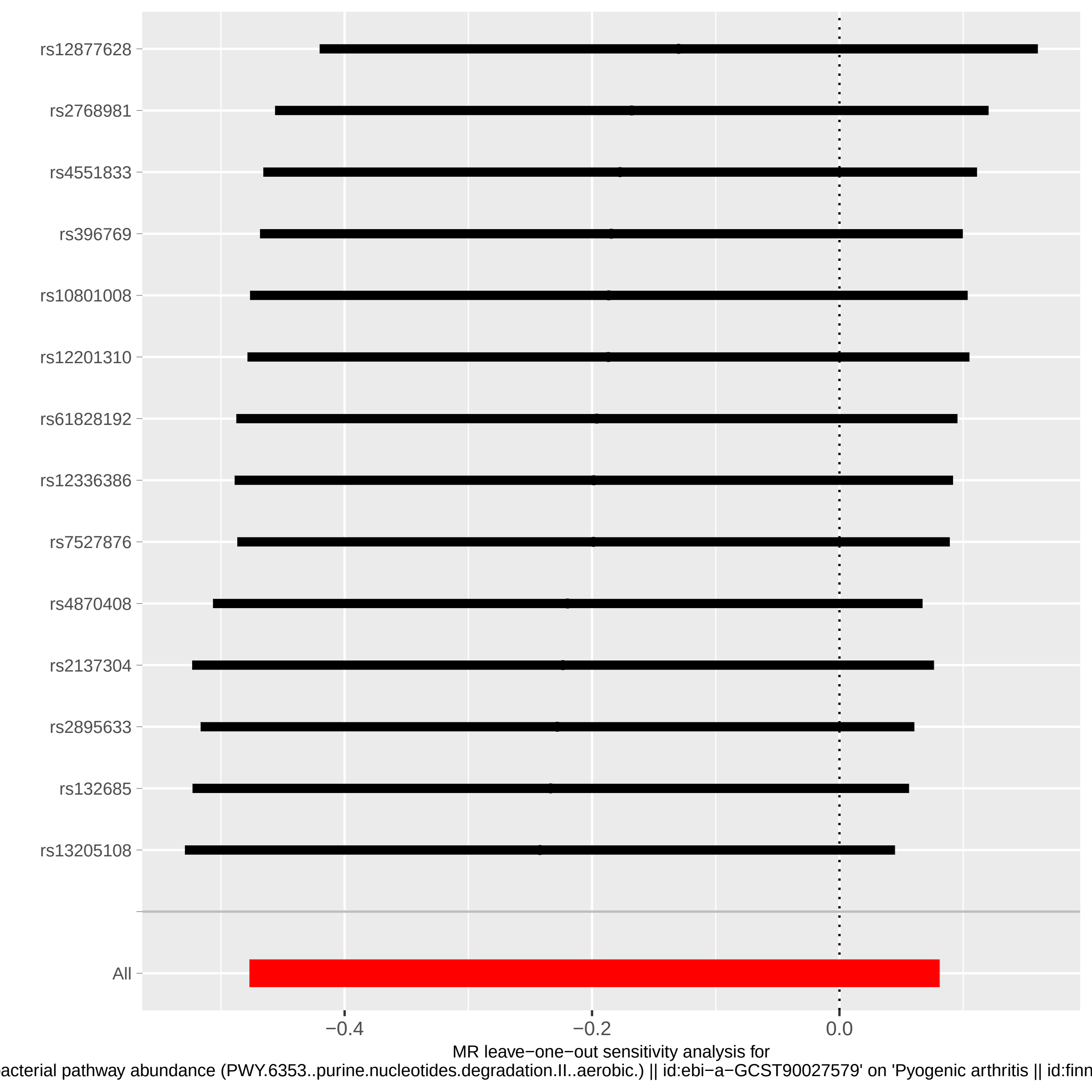
rs12877628
rs2768981
rs4551833
rs396769
rs10801008
rs12201310
rs61828192
rs12336386
rs7527876
rs4870408
rs2137304
rs2895633
rs132685
rs13205108
All
−0.4
−0.2
0.0
MR leave−one−out sensitivity analysis for
acterial pathway abundance (PWY.6353..purine.nucleotides.degradation.II..aerobic.) || id:ebi−a−GCST90027579' on 'Pyogenic arthritis || id:finn
